# Association between triglyceride-glucose index and risk of arterial stiffness: a cohort study

**DOI:** 10.1186/s12933-021-01342-2

**Published:** 2021-07-16

**Authors:** Shouling Wu, Luli Xu, Mingyang Wu, Shuohua Chen, Youjie Wang, Yaohua Tian

**Affiliations:** 1 Department of Cardiology, Kailuan General Hospital, North China University of Science and Technology, No.57 Xinhua East Street, Tangshan, 063000 China; 2 Department of Maternal and Child Health, School of Public Health, Tongji Medical College, Huazhong University of Science and Technology, No.13 Hangkong Road, Wuhan, 430030 China; 3 Ministry of Education Key Laboratory of Environment and Health, and State Key Laboratory of Environmental Health (Incubating), School of Public Health, Tongji Medical College, Huazhong University of Science and Technology, No.13 Hangkong Road, Wuhan, 430030 China

**Keywords:** Triglyceride glucose index, Arterial stiffness, Brachial-ankle pulse wave velocity, Insulin resistance, Cohort study

## Abstract

**Background:**

Triglyceride–glucose (TyG) index, a simple surrogate marker of insulin resistance, has been reported to be associated with arterial stiffness. However, previous studies were limited by the cross-sectional design. The purpose of this study was to explore the longitudinal association between TyG index and progression of arterial stiffness.

**Methods:**

A total of 6028 participants were derived from the Kailuan study. TyG index was calculated as ln [fasting triglyceride (mg/dL) × fasting glucose (mg/dL)/2]. Arterial stiffness was measured using brachial-ankle pulse wave velocity (baPWV). Arterial stiffness progression was assessed by the annual growth rate of repeatedly measured baPWV. Multivariate linear regression models were used to estimate the cross-sectional association of TyG index with baPWV, and Cox proportional hazard models were used to investigate the longitudinal association between TyG index and the risk of arterial stiffness.

**Results:**

Multivariate linear regression analyses showed that each one unit increase in the TyG index was associated with a 39 cm/s increment (95%CI, 29–48 cm/s, *P* < 0.001) in baseline baPWV and a 0.29 percent/year increment (95%CI, 0.17–0.42 percent/year, *P* < 0.001) in the annual growth rate of baPWV. During 26,839 person-years of follow-up, there were 883 incident cases with arterial stiffness. Participants in the highest quartile of TyG index had a 58% higher risk of arterial stiffness (HR, 1.58; 95%CI, 1.25–2.01, *P* < 0.001), as compared with those in the lowest quartile of TyG index. Additionally, restricted cubic spline analysis showed a significant dose–response relationship between TyG index and the risk of arterial stiffness (*P* non-linearity = 0.005).

**Conclusion:**

Participants with a higher TyG index were more likely to have a higher risk of arterial stiffness. Subjects with a higher TyG index should be aware of the following risk of arterial stiffness progression, so as to establish lifestyle changes at an early stage.

**Supplementary Information:**

The online version contains supplementary material available at 10.1186/s12933-021-01342-2.

## Background

Cardiovascular disease (CVD) has climbed to the first cause of death and premature death in China [1, 2]. Arterial stiffness, one of the earliest functional damage in the vascular aging process, directly influenced the cardiovascular system by degenerating arterial elasticity and increasing pulse pressure [3, 4]. A growing number of studies demonstrated that arterial stiffness was a predictor for future cardiovascular events such as myocardial infarction, unstable angina, heart failure, and ischemic or hemorrhagic stroke [5, 6]. Considering that the pathology of arterial stiffness is a long-term progression, there is an urgent need for available and reliable markers to identify patients in the early stage and formulate appropriate preventive strategies.

Insulin resistance (IR) is regarded as an important contributing factor for arterial stiffness and the development of CVD due to the production of inflammatory factors and the following endothelial damage [7–9]. The triglyceride–glucose (TyG) index, which is calculated from fasting blood glucose (FBG) and fasting triglyceride (TG) levels, has been proposed as a surrogate marker of IR with better performance than the homeostasis model assessment of IR (HOMA-IR) [10–12]. Some previous studies have indicated a positive association of TyG index with cardiovascular risk including coronary artery disease, stroke, myocardial infarction, and carotid atherosclerosis [12–17]. Recently, a growing number of studies have been conducted to investigate the relationship between TyG index and arterial stiffness [11, 15, 17–20]. However, most previous studies were conducted with a cross-sectional study design, but not a cohort study on the progression of arterial stiffness. Therefore, we extend previous findings and provide a longitudinal perspective to explore the association between TyG index and the progression of arterial stiffness measured using brachial-ankle pulse wave velocity (baPWV).

## Methods

### Study population

The participants were derived from Kailuan cohort, a prospective cohort established at Kailuan General Hospital and 10 affiliated hospitals, which has been described in detail elsewhere [21]. Briefly, from June 2006 to October 2007, participants from Kailuan community were enrolled and completed the first survey including questionnaires, physical examinations, and laboratory assessments, with an every 2-year follow-up. Since the third survey in 2010, a subcohort of the nested study on vascular health was conducted, with the baPWV tests to assess the health status of artery wall [22]. In this study, we included participants according to the following criteria: (1) individuals participated in at least one survey in 2010–2011, 2012–2013, 2014–2015, or 2016–2017, and received the first baPWV test; (2) individuals had complete baseline data of FBG and TG. There were 34,099 subjects who met the criteria. Then, we excluded participants who: (1) were taking antidiabetic or antihyperlipidemic agents at baseline; (2) had a history of CVD or cancer at baseline; (3) had baPWV tests less than three. Finally, a total of 6028 participants were included for analysis.

The study was performed according to the guidelines of the Declaration of Helsinki and was approved by the Ethics Committee of the Kailuan General Hospital (Approval Number: 2006–05). Written informed consent was obtained from all participants.

### Data collection and definition

Information on demographics (e.g., age, sex), medical history (e.g., medications), and lifestyle (e.g., smoking status, alcohol consumption, physical activity), was collected by a structural questionnaire. Smoking and drinking status were stratified into two levels: current and never or former. Active physical activity was defined as “≥ 80 min/week”. Anthropometric data including weight, height, and waist circumference was measured by trained staff. Body mass index (BMI) was defined as the weight (kg)/height (m)^2^. Blood pressure was measured in the seated position using a mercury sphygmomanometer, and the average of three readings was calculated as systolic blood pressure (SBP) and diastolic blood pressure (DBP). Mean arterial blood pressure (MAP) was defined as 1/3*SBP + 2/3*DBP [23]. Hypertension was defined as either SBP or DBP ≥ 140/90 mmHg, antihypertensive medication use, or a self-reported history of hypertension. Diabetes was defined as FBG ≥ 7.0 mmol/L or self-reported history of diabetes.

Blood samples were collected after fasting for 8 to 12 h. Biochemical parameters including FBG, TG, total cholesterol (TC), high-density lipoprotein (HDL), low-density lipoprotein (LDL), and high-sensitivity C-reactive protein (hs-CRP) were measured on the Hitachi 747 auto-analyzer (Hitachi, Tokyo, Japan). FBG and TG levels were measured by the hexokinase/glucose-6-phosphate dehydrogenase method and the enzymatic colorimetric method, respectively. TyG index was calculated as ln [fasting triglyceride (mg/dL) × fasting glucose (mg/dL)/2] [24].

### BaPWV measurement

BaPWV was measured by a BP-203 RPE III networked arterial stiffness detection device (Omron Health Medical [China]Co., LTD), as detailed elsewhere [22, 25, 26]. Measurements were repeated twice for each participant at a 5 min interval, with the second data considered the final value. The larger value between the left and right sides was used for analysis. BaPWV ≥ 1800 cm/s was considered as arterial stiffness according to the previous studies [27]. The progression of arterial stiffness was appraised by the annual growth rate of baPWV, which was calculated as

$$\sum_{di=d2}^{dn}\left({baPWV}_{{1}^{*}}{\overline{r} }^{di}\right)=\sum_{i=2}^{n}{baPWV}_{i}$$

$i, d2, di, {baPWV}_{1},{baPWV}_{i}\,and\, \overline{r }$ represented the survey ordinal number, the duration from baseline to the second survey, the duration from baseline to the ${i}_{th}$ survey, the baseline baPWV, the value of baPWV at the ${i}_{th}$ survey, and annual growth rate of baPWV, respectively.

### Statistical analysis

Continuous variables with normal and skewed distributions were described as the mean ± standard deviation (SD) and the medians with interquartile ranges, respectively, whereas categorical variables were described as frequencies and percentages. Missing data for baseline covariates were imputed by the multiple imputation. To investigate linear trends of baseline characteristics across quartiles of TyG index, chi-square test for linear trend was used for categorical variables, and one-way analysis of variance for linear trend and Kruskal–Wallis test was performed for continuous variables with normal and skewed distributions, respectively. The TyG index was assessed by both quartiles and continuous variables. The associations of TyG index with baPWV at baseline and the annual growth rate of baPWV were estimated using multivariate linear regression models. Covariates referred to factors significantly associated with the progression of arterial stiffness and were ascertained according to prior published literature [23, 28], including age, sex, physical activity, smoking, alcohol drinking, BMI, hs-CRP, diabetes, and MAP.

Additionally, to investigate the association between TyG index and the risk of arterial stiffness, 680 participants with baPWV ≥ 1800 cm/s at baseline were excluded from the dataset. The remaining 5348 subjects without arterial stiffness were followed up to Jul 2020. Multivariate Cox proportional hazard regression was used to estimate the adjusted hazard ratios (HRs) and 95% confidence intervals  (95%CIs) for arterial stiffness across the quartiles of the TyG index. In addition, we carried out restricted cubic spline analysis to explore the dose–response relationship between TyG index and baPWV as well as the risk of arterial stiffness. Three knots were placed at the 10th, 50th, and 90th percentiles, and the median of TyG index was used for reference point.

To avoid the potential bias due to measurement frequency, the associations between TyG index and the progression of arterial stiffness were reanalyzed among participants with at least two baPWV tests (N = 12,706), and the results were presented in Supplement materials as a sensitivity analysis. We also presented baseline characteristics of those with at least two baPWV tests according to the quartiles of the TyG index, and shown these overall characteristics for participants with only two baPWV tests and those with at least three baPWV tests in Supplement materials. All statistical analyses were performed using R software (version 4.0.2), and a two-tailed *P* value < 0.05 was considered statistically significant.

## Results

### Baseline characteristics

A total of 34,099 participants received baPWV tests with complete data of FBG and TG. After a series of exclusion criteria, 6028 individuals were included in the present study (Additional file 1: Fig. S1). Clinical and laboratory characteristics at baseline are presented in Table 1 according to the quartiles of the TyG index. Compared with participants in the lowest quartile group, participants with a higher TyG index tended to be older, men, more obese with higher BMI and waist circumference, and current smokers or drinkers. Likewise, significant differences in biological parameters were observed among the groups. SBP, DBP, MAP, FPG, TC, TG, LDL, and hs-CRP of the participants in the highest TyG index quartile were significantly higher than those in the first quartile. Moreover, the high TyG index groups had a higher prevalence of comorbidities such as diabetes and hypertension.

**Table 1:** Baseline characteristics of participants according to quartiles of TyG index

Characteristics	Quartiles of TyG index				*P* for trend
	Q1(6.81–8.10)	Q2(8.10–8.52)	Q3(8.52–9.02)	Q4(9.02–13.17)	
Age, years^a^	43.6 ± 11.0	47.0 ± 12.1	49.3 ± 12.9	48.1 ± 12.4	< 0.001
Male, n (%)^b^	484 (32.2)	750 (49.7)	907 (60.2)	1040 (69.0)	< 0.001
Active physical activity, n (%)^b^	231 (15.3)	237 (15.7)	291 (19.3)	262 (17.4)	0.025
Current smoker, n (%)^b^	245 (16.3)	378 (25.0)	448 (29.7)	541 (35.9)	< 0.001
Current alcohol use, n (%)^b^	206 (13.7)	290 (19.2)	344 (22.8)	448 (29.7)	< 0.001
BMI, kg/m^2 a^	23.1 ± 3.1	24.3 ± 3.1	25.2 ± 3.4	26.3 ± 3.4	< 0.001
Waist circumference, cm ^c^	78 (72–85)	83 (76–89)	86 (80–92)	90 (83–96)	< 0.001
FBG, mmol/L^c^	4.9 (4.5–5.2)	5.1 (4.7–5.4)	5.2 (4.9–5.7)	5.6 (5.1–6.3)	< 0.001
SBP, mmHg ^c^	116.7 (105.0–128.7)	120.0 (110.0–131.7)	129.3 (117.7–140.0)	130.0 (120.0–140.0)	< 0.001
DBP, mmHg ^c^	77.0 (70.0–81.3)	80.0 (71.3–87.0)	80.7 (76.7–90.0)	84.0 (80.0–90.3)	< 0.001
MAP, mmHg ^c^	90.0 (81.3–96.7)	93.3 (86.7–101.7)	96.7 (90.0–106.0)	100.0 (93.3–108.2)	< 0.001
TC, mmol/L^c^	4.4 (4.0–5.0)	4.8 (4.2–5.3)	5.1 (4.5–5.7)	5.3 (4.7–6.0)	< 0.001
TG, mmol/L^c^	0.6 (0.6–0.8)	1.0 (0.9–1.1)	1.5 (1.3–1.7)	2.7 (2.1–3.8)	< 0.001
HDL, mmol/L^c^	1.6 (1.4–1.9)	1.5 (1.3–1.8)	1.4 (1.2–1.8)	1.3 (1.1–1.6)	< 0.001
LDL, mmol/L^c^	2.2 (1.8–2.6)	2.5 (2.1–3.0)	2.7 (2.2–3.2)	2.7 (2.1–3.3)	< 0.001
TyG index^a^	7.8 ± 0.2	8.3 ± 0.1	8.7 ± 0.1	9.6 ± 0.5	< 0.001
hs-CRP^a^	1.6 ± 3.7	1.7 ± 2.9	2.2 ± 4.5	2.3 ± 3.2	< 0.001
baPWV, cm/s ^a^	1301.1 ± 266.5	1399.8 ± 276.5	1491.5 ± 323.1	1540.1 ± 324.7	< 0.001
Diabetes, n (%)^b^	2 (0.1)	15 (1.0)	59 (3.9)	258 (17.1)	< 0.001
Hypertension, n (%)^b^	263 (17.5)	440 (29.2)	646 (42.9)	771 (51.2)	< 0.001

N = 6028; *Q* quartiles, *BMI* body mass index, *FBG* fasting blood glucose, *SBP* systolic blood pressure, *DBP* diastolic blood pressure;, *MAP* mean arterial blood pressure, *TC* total cholesterol, *TG* triglyceride, *HDL* high-density lipoprotein, *LDL* low-density lipoprotein, *TyG index* triglyceride–glucose index, *hs-CRP* high-sensitivity C-reactive protein, *baPWV* brachial-ankle pulse wave velocity; ^a^mean ± standard deviation, and the variables were tested by one-way analysis of variance for linear trend; ^b^the variables were tested by chi-square test for linear trend; ^c^median (interquartile range), and the variables were tested by Kruskal–Wallis test

### TyG index and baPWV

**Fig. 1:**
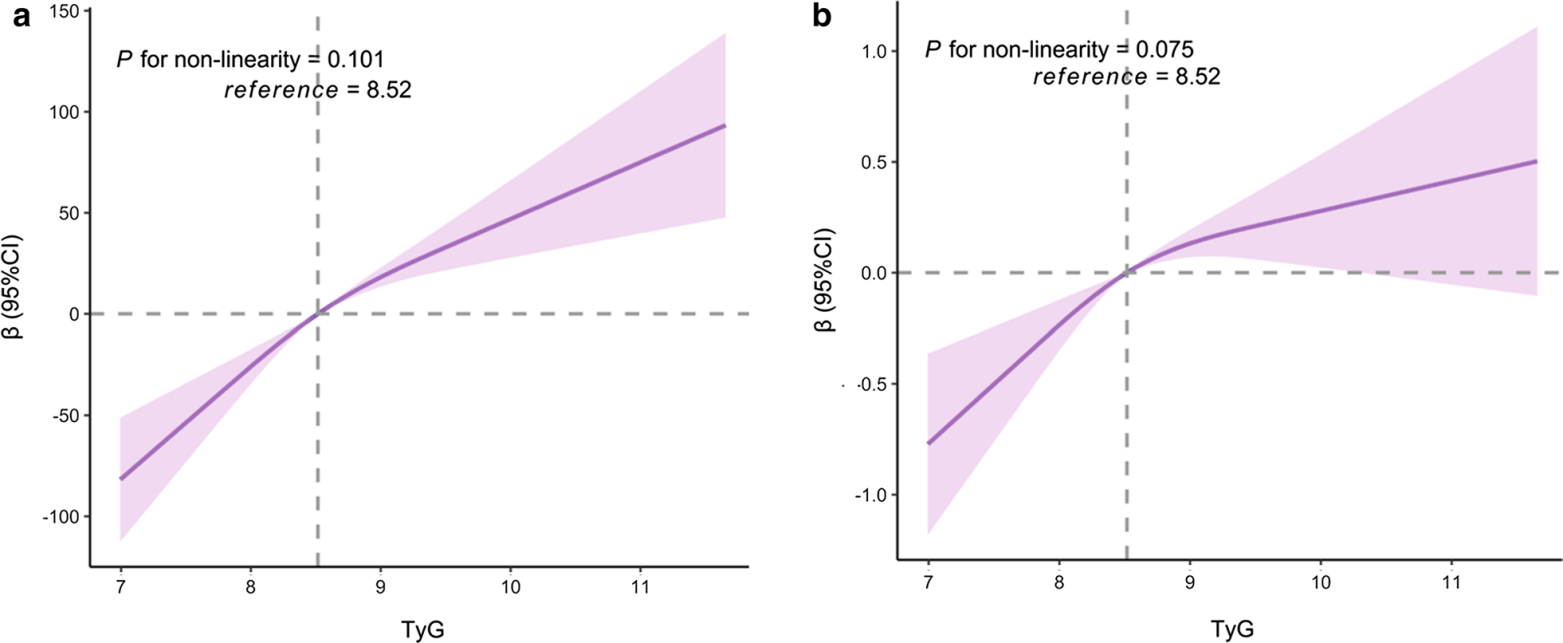
The associations of TyG index with baPWV at baseline (**a**) and annual growth rate of baPWV (**b**). Data were fitted using the linear regression models of the restricted cubic spline with 3 knots at 10, 50, and 90th percentiles of baseline TyG index. *TyG index* triglyceride-glucose index,*CI* confidence interval. The reference point was the median of the TyG index in the 6028 participants. The solid line represented point estimation on the association of TyG index with baPWV, and the shaded portion represented 95% CI estimation. Covariates in the model included age, sex, smoking, alcohol drinking, physical activity, MAP, diabetes, hs-CRP, and BMI

Overall, there was a significant positive association of TyG index with baPWV at baseline (Table 2, Fig. 1a). The linear regression models revealed that each one unit increment of TyG index was associated with a 39 cm/s change in baPWV (95% CI, 29–48 cm/s, *P* < 0.001) after adjusting for age, sex, physical activity, smoking, alcohol drinking, BMI, hs-CRP, diabetes, and MAP. In the categorical analysis, compared with those in the first quartile of TyG index, the adjusted *β* for participants in the second, third and highest quartile of TyG index were 19 (95% CI, 2–36 cm/s, *P* = 0.032), 50 (95% CI, 32–67 cm/s, *P* < 0.001), and 71 (95% CI, 52–90 cm/s, *P* < 0.001), respectively.

**Table 2:** Association of TyG index with baPWV at baseline and baPWV progression in linear models

TyG index	Model 1		Model 2	
	*β*(95% CI)	*P* value	*β*(95% CI)	*P* value
baPWV at baseline				
Per 1 unit increase	75 (66–85)	< 0.001	39 (29–48)	< 0.001
Q1 (6.81–8.10)	Reference		Reference	
Q2 (8.10–8.52)	36 (18–55)	< 0.001	19 (2–36)	0.032
Q3 (8.52–9.02)	88 (69–110)	< 0.001	50 (32–67)	< 0.001
Q4 (9.02–13.17)	140 (120–160)	< 0.001	71 (52–90)	< 0.001
baPWV progression				
Per 1 unit increase	0.42 (0.31–0.54)	< 0.001	0.29 (0.17–0.42)	< 0.001
Q1 (6.81–8.10)	Reference		Reference	
Q2 (8.10–8.52)	0.41 (0.19–0.64)	< 0.001	0.34 (0.11–0.57)	0.003
Q3 (8.52–9.02)	0.67 (0.44–0.91)	< 0.001	0.52 (0.29–0.76)	< 0.001
Q4 (9.02–13.17)	0.72 (0.48–0.96)	< 0.001	0.45 (0.20–0.71)	< 0.001

N = 6028; TyG index, triglyceride–glucose index; baPWV, brachial-ankle pulse wave velocity; CI, confidence interval; Q, quartiles

Model 1, adjusted for age and sex at baseline

Model 2, adjusted for variables in model 1 plus smoking, alcohol drinking, physical activity, MAP, diabetes, hs-CRP, and BMI at baseline

We further assessed the association of the TyG index with the annual growth rate of baPWV and found that the growth rate of baPWV had a statically significant increment in participants with a high TyG index (Fig. 1b). Overall, each one unit increase in the TyG index was responsible for a 0.29 percent/year increment in the average growth rate of baPWV after adjusting for all covariates (95% CI, 0.17–0.42 percent/year, *P* < 0.001). When TyG index was assessed as quartiles, as compared with those in the first quartile, *β* for participants in the second, third, and highest quartile of TyG index were 0.34 (95% CI, 0.11–0.57 percent/year, *P* = 0.003), 0.52 (95% CI, 0.29–0.76 percent/year, *P* < 0.001), and 0.45 (95% CI, 0.20–0.71 percent/year, *P* < 0.001) in the fully adjusted model, respectively.

### TyG index and arterial stiffness

**Fig. 2:**
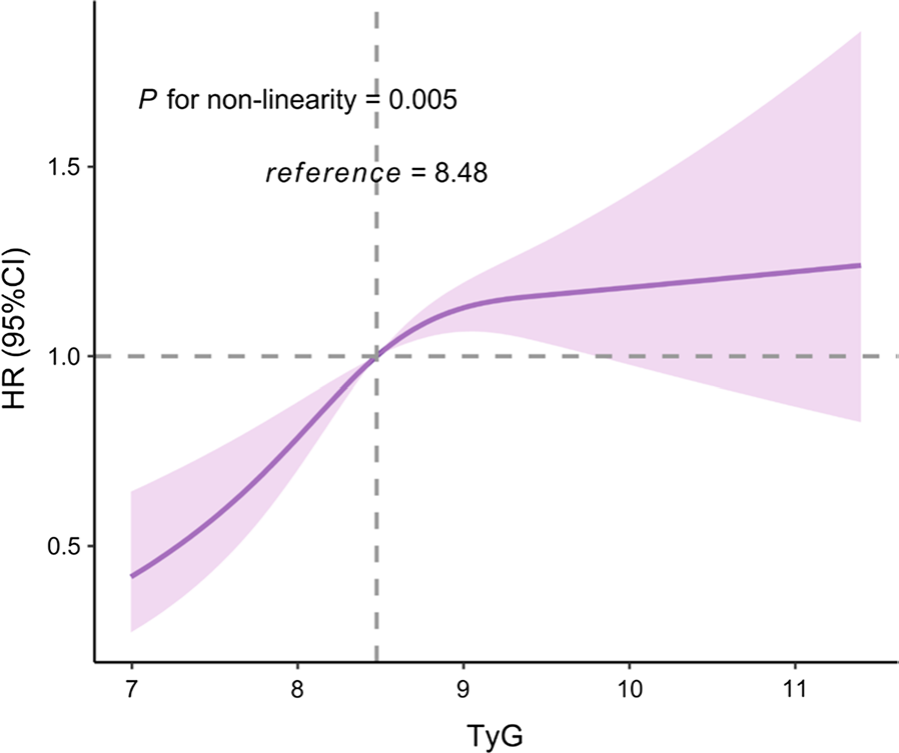
The associations of TyG index with risk of arterial stiffness. Data were fitted using a Cox regression model of the restricted cubic spline with 3 knots at 10th, 50th, and 90th percentiles of baseline TyG index. *TyG index* triglyceride-glucose index, *HR* hazard ratio, *CI* confidence interval. The reference point was the median of the TyG index in the 5348 participants. The solid line represented point estimation on the association of TyG index with the risk of arterial stiffness, and the shaded portion represented 95% CI estimation. Covariates in the model included age, sex, smoking, alcohol drinking, physical activity, MAP, diabetes, hs-CRP, and BMI

During 26,839 person-years of follow-up, there were 883 incident cases of arterial stiffness among the 5348 participants. The incident rate presented an increasing trend in four groups divided by the quartiles of TyG index, which were 14.46, 26.21, 44.78, and 49.87 per 1000 person-years, respectively. The association between the baseline TyG index and the risk of arterial stiffness is presented in Table 3. After adjusting for all covariates, the risk for arterial stiffness increased 22% (95% CI, 1.10–1.35, *P* < 0.001) with each one-unit increase of TyG index. Taking the lowest quartile as a reference, the adjusted HRs (95% CIs) in the second, third and highest quartiles of TyG index were 1.27 (1.00–1.62), 1.69 (1.35–2.12), and 1.58 (1.25–2.01), respectively. In addition, restricted cubic spline analysis (Fig. 2) showed the dose–response relationship between TyG index and the risk of arterial stiffness. (*P* non-linearity = 0.005).

**Table 3:** Association of TyG index with arterial stiffness in Cox proportional hazard models

TyG index	Arterial stiffness(n)	Incident rate (per 1000 person-years)	Model 1		Model 2	
			HR (95%CI)	*P* value	HR (95%CI)	*P* value
Overall	883	32.90	1.46 (1.34–1.58)	< 0.001	1.22 (1.10–1.35)	< 0.001
Q1 (6.81–8.06)	106	14.46	Reference		Reference	
Q2 (8.06–8.48)	181	26.21	1.39 (1.09–1.77)	0.008	1.27 (1.00–1.62)	0.052
Q3 (8.48–8.98)	285	44.78	2.05 (1.64–2.57)	< 0.001	1.69 (1.35–2.12)	< 0.001
Q4 (8.98–13.17)	311	49.87	2.30 (1.84–2.87)	< 0.001	1.58 (1.25–2.01)	< 0.001

N = 5348; TyG index, triglyceride–glucose index; HR, hazard ratio; CI, confidence interval; Q, quartiles

Model 1, adjusted for age and sex at baseline

Model 2, adjusted for variables in model 1 plus smoking, alcohol drinking, physical activity, MAP, diabetes, hs-CRP, and BMI at baseline

### Sensitivity analysis

The baseline characteristics of participants with only two baPWV tests and at least three baPWV tests was shown in Additional file 2: Table S1. Overall, the characteristics between the two groups were similar. In addition, the results of sensitivity analyses showed similar significant associations between TyG index and the progression of arterial stiffness among participants with at least two baPWV tests. (Additional file 2: Table S2–S4).

## Discussion

In this study, we identified a significant association of the TyG index with baPWV as a quantitative marker of arterial stiffness. The TyG index was related to baPWV at baseline as well as the annual growth rate of baPWV in form of both quartiles and continuous variables. Furthermore, the TyG index was observed to be associated with the incidence of arterial stiffness assessed by baPWV after adjusting for age, sex, smoking, alcohol drinking, physical activity, MAP, diabetes, hs-CRP, and BMI at baseline. To the best of our knowledge, this is the first study to reveal the relationship between the TyG index and the progression of arterial stiffness in a large prospective cohort.

Recently, quite a few cohort studies have confirmed that the TyG index could play a predictive role in adverse cardiovascular events [14, 29–32]. Furthermore, several researchers transferred their attention to the association of TyG index with the progression of arterial-related pathology, particularly coronary artery calcium (CAC) and coronary plaque progression (PP). Two studies in Korea suggested that elevated TyG index was associated with a higher risk for CAC progression [16, 33]. In addition, Won et al*.* reported that the participants with the highest tertile TyG index had a 1.65-fold higher risk of coronary PP and 1.78-fold of rapid PP compared with the lowest tertile [34]. However, to our knowledge, little is currently known about the relationship between TyG index and arterial stiffness progression. Previous researchers have explored a cross-sectional association of TyG index with arterial stiffness assessed by baPWV. For instance, Lee and his colleagues found a nearly three-fold higher risk of increased baPWV in men and two-fold in women for the highest quartiles TyG index compared with the lowest quartiles in a general population [11]. Similarly, Won et al*.* demonstrated a linear relationship between the TyG index and the baPWV in both non-diabetics and diabetics [18]. This association was also observed in hypertensive patients [17]. Consistent with the abovementioned studies, findings from the present study also showed a significant cross-sectional association between TyG index and increased baPWV. We further investigate the longitudinal relationship of TyG index with the risk of arterial stiffness progression assessed by repeated measurement of baPWV, and found that a higher TyG index predicted a higher risk and faster progression of arterial stiffness. These findings extended the limited available evidence on the association between TyG index and arterial stiffness and may be helpful for early identification of subjects with a high risk of arterial stiffness, so as to take appropriate strategies to prevent CVD.

Although the exact biological mechanisms underlying the association remain unclear, the possible crucial pathway may be linked to IR. Generally, IR perturbs insulin signaling at the level of the intimal cells including endothelial cells, vascular smooth muscle cells, and macrophages, leading to a varying degree of oxidative responses and impaired endothelial function [35–37]. Moreover, IR drives the development of atherogenic dyslipidemia, and generates a low­grade inflammatory state [7]. Previous researches have proved that inflammation was a key biological process involved in the pathogenesis of arterial stiffness, and it was often marked by CRP [8, 23, 37]. In our study, we observed that people with the highest quartile TyG index had a higher level of hs-CRP than those in the first quartile group. Nevertheless, the association of TyG index with arterial stiffness was still significant after adjustment for hs-CRP. This indicated an independent effect of TyG index on arterial stiffness progression. Due to relatively limited studies on this issue so far, more studies are warranted to be conducted for elucidating the exact pathways.

Simental-Mendía et al*.* firstly introduced TyG index as a surrogate to identify IR, and suggested the higher sensitivity of TyG index than HOMA-IR [24]. Subsequently, they also reported that TyG index closely mirrors the euglycemic-hyperinsulinemic clamp test, the gold standard technique in the assessment of insulin sensitivity [38]. In our study, we compared the diagnostic values of the TyG index, FBG, and TG for arterial stiffness by receiver operating characteristics curve and the area under the curve, and found that TyG index had a better predictive value than FBG and TG alone (data not shown), which was consistent with previous studies [39–41]. With an increasing number of studies supporting the strong predictive value of TyG index for the development of CVDs and other chronic diseases such as type 2 diabetes [42, 43], TyG index has been widely recognized as a simple and effective marker for disease prediction and prevention. A high TyG index could alert people to establish early lifestyle changes able to reduce the progression or incidence of disease.

Our study has several limitations. First, although most available demographic and clinical variables were incorporated into the model for adjustment, some residual or unmeasured confounding parameters such as the dose of alcoholic beverages and the consumption of macronutrients and nutrients could have affected the results. Second, it should be noted that the participants in this study were all from the Kailuan community, thus the findings may not be generalizable to other populations. Third, owing to a lack of records on serum insulin, we could not compare the TyG index with HOMA-IR and the hyperinsulinemic-euglycemic clamp test. Fourth, as a long-term cohort study, there existed an unavoidable bias caused by loss to follow-up. However, the sensitivity analysis showed that the associations between TyG index and the progression of arterial stiffness remained in the participants with at least two baPWV tests.

## Conclusions

In conclusion, participants with a higher TyG index were more likely to have a higher risk of arterial stiffness. Subjects with a higher TyG index should be aware of the following risk of arterial stiffness progression, so as to establish lifestyle changes at an early stage.

## Supplementary Information

**Additional file 1: Fig. S1.** Flow chart of study population.

**Additional file 2: Table S1.** Baseline characteristics of participants with only two and at least three baPWV tests**. Table S2.** Baseline characteristics of participants with at least two baPWV tests**. Table S3.** Association of TyG index with baPWV in participants with at least two baPWV tests**. Table S4.** Association of TyG index with arterial stiffness in participants with at least two baPWV tests.

## Data Availability

The datasets used and/or analyzed during the current study are available from the corresponding author on reasonable request.

## Abbreviations

CVD: Cardiovascular disease
TyG index: Triglyceride-glucose index
IR: Insulin resistance
HOMA-IR: Homeostasis model assessment of insulin resistance
baPWV: Brachial-ankle pulse wave velocity
BMI: Body mass index
SBP: Systolic blood pressure
DBP: Diastolic blood pressure
MAP: Mean arterial blood pressure
FBG: Fasting blood glucose
TG: Triglyceride
TC: Total cholesterol
HDL: High-density lipoprotein
LDL: Low-density lipoprotein
hs-CRP: High-sensitivity C-reactive protein
SD: Standard deviation
HR: Hazard ratio
CI: Confidence interval
CAC: Coronary artery calcium
PP: Plaque progression
