# Supplementary material for: Association between triglyceride-glucose index and risk of arterial stiffness: a cohort study
Source: Cardiovasc Diabetol. 2021 Jul 16;20:146. doi: 10.1186/s12933-021-01342-2 (PMC8285795; doi:10.1186/s12933-021-01342-2)
Supplement: Supplementary file 1 — Additional file 1: Fig. S1. Flow chart of study population. [file 12933_2021_1342_MOESM1_ESM.docx]

**Additional File 1**

**Additional figure**

**Fig. S1** Flow chart of study population.


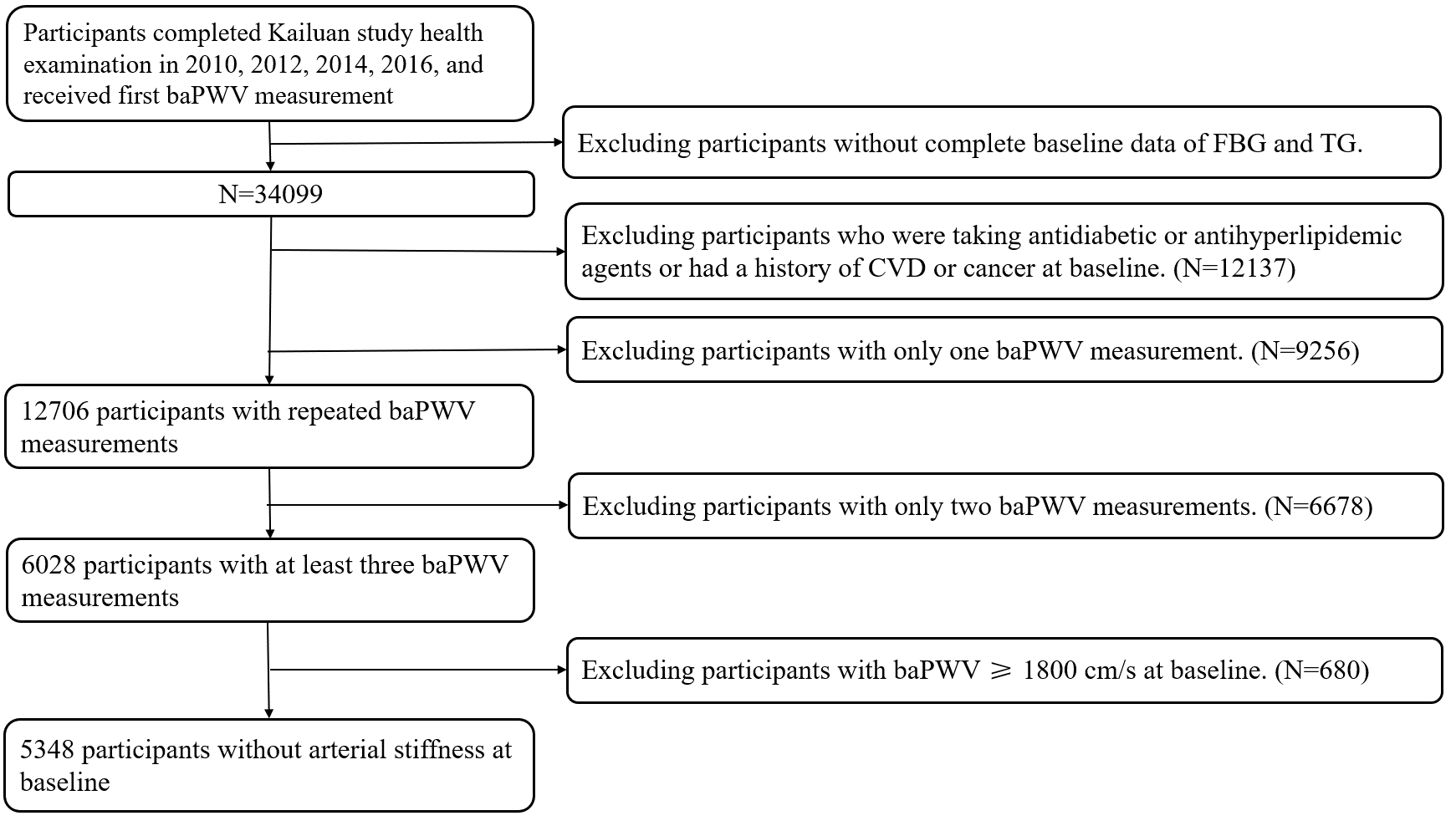


**Fig. S1** Flow chart of study population.
