# Supplementary material for: Association between triglyceride-glucose index and risk of arterial stiffness: a cohort study
Source: Cardiovasc Diabetol. 2021 Jul 16;20:146. doi: 10.1186/s12933-021-01342-2 (PMC8285795; doi:10.1186/s12933-021-01342-2)
Supplement: Supplementary file 2 — Additional file 2: Table S1. Baseline characteristics of participants with only two and at least three baPWV tests. Table S2. Baseline characteristics of participants with at least two baPWV tests. Table S3. Association of TyG index with baPWV in participants with at least two baPWV tests. Table S4. Association of TyG index with arterial stiffness in participants with at least two baPWV tests. [file 12933_2021_1342_MOESM2_ESM.docx]

**Additional File 2**

**Additional tables**

**Table S1.** Baseline characteristics of participants with only two and at least three baPWV tests

**Table S2.** Baseline characteristics of participants with at least two baPWV tests (N= 12706)

**Table S3** Association of TyG index with baPWV in participants with at least two baPWV tests (N=12706)

**Table S4** Association of TyG index with arterial stiffness in participants with at least two baPWV tests

| **Table S1** Baseline characteristics of participants with only two and at least three baPWV tests | | |
| --- | --- | --- |
| Characteristics | Participants with only two baPWV tests (N=6678) | Participants with at least three baPWV tests (N=6028) |
| Age, years | 47.3±13.9 | 47.0±12.3 |
| Sex, male | 4738 (70.9) | 3181 (52.8) |
| Active physical activity, n (%) | 1429 (21.4) | 1036 (17.2) |
| Current smoker, n (%) | 2468 (37.0) | 1612 (26.7) |
| Current alcohol use, n (%) | 1074 (16.1) | 1287 (21.4) |
| BMI, kg/m^2^ | 24.9±3.4 | 24.7±3.4 |
| Waist circumference, cm | 86 (80-92) | 84 (77-90) |
| FBG, mmol/L | 5.2 (4.8-5.8) | 5.1 (4.8-5.6) |
| SBP, mmHg | 130.0 (119.3-140.0) | 123.3 (111.0-138.0) |
| DBP, mmHg | 80.7 (75.0-90.0) | 80.0 (72.3-90.0) |
| MAP, mmHg | 96.7 (90.0-105.7) | 94.9 (86.7-103.3) |
| TC, mmol/L | 4.9 (4.3-5.6) | 4.9 (4.3-5.5) |
| TG, mmol/L | 1.3 (0.9-2.0) | 1.2 (0.8-1.9) |
| HDL, mmol/L | 1.4 (1.2-1.6) | 1.5 (1.2-1.8) |
| LDL, mmol/L | 2.7 (2.2-3.2) | 2.5 (2.0-3.0) |
| TyG index | 8.7±0.7 | 8.6±0.7 |
| hs-CRP | 2.0±4.1 | 2.0±3.6 |
| baPWV, cm/s | 1488.3±332.2 | 1433.1±312.5 |
| Diabetes, n (%) | 510 (7.6) | 334 (5.5) |
| Hypertension, n (%) | 2504 (37.5) | 2120 (35.2) |
| Note: BMI, body mass index; FBG, fasting blood glucose; SBP, systolic blood pressure; DBP, diastolic blood pressure; MAP, mean arterial blood pressure; TC, total cholesterol; TG, fast triglyceride; HDL, high-density lipoprotein; LDL, low-density lipoprotein; TyG index, triglyceride–glucose index; hs-CRP, high-sensitivity C-reactive protein; baPWV, brachial-ankle pulse wave velocity; | | |

| **Table S2** Baseline characteristics of participants with at least two baPWV tests (N= 12706) | | | | | |
| --- | --- | --- | --- | --- | --- |
| Characteristics | Quartiles of TyG index | | | | *P* for trend |
|  | Q1(5.88-8.14) | Q2(8.14-8.56) | Q3(8.56-9.06) | Q4(9.06-13.17) |  |
| Age, years ^a^ | 44.2±12.7 | 47.3±13.2 | 48.9±13.5 | 48.4±12.7 | <0.001 |
| Male, n (%) ^b^ | 1442 (45.4) | 1932 (60.9) | 2188 (68.9) | 2357 (74.2) | <0.001 |
| Active physical activity, n (%) ^b^ | 549 (17.3) | 588 (18.5) | 662 (20.8) | 666 (21.0) | <0.001 |
| Current smoker, n (%) ^b^ | 731 (23.0) | 974 (30.7) | 1097 (34.5) | 1278 (40.2) | <0.001 |
| Current alcohol use, n (%) ^b^ | 412 (13.0) | 556 (17.5) | 640 (20.1) | 753 (23.7) | <0.001 |
| BMI, kg/m^2 a^ | 23.1±3.1 | 24.4±3.1 | 25.3±3.2 | 26.3±3.4 | <0.001 |
| Waist circumference, cm ^c^ | 80 (73-86) | 84 (78-90) | 87 (81-93) | 90 (84-96) | <0.001 |
| FBG, mmol/L ^c^ | 4.9 (4.6-5.3) | 5.1 (4.8-5.5) | 5.3 (4.9-5.8) | 5.6 (5.1-6.5) | <0.001 |
| SBP, mmHg ^c^ | 120.0 (109.3-130.0) | 123.3 (114.0-136.3) | 130.0 (120.0-140.0) | 131.7 (120.7-144.0) | <0.001 |
| DBP, mmHg ^c^ | 78.0 (70.0-82.0) | 80.0 (72.7-88.0) | 81.3 (78.0-90.0) | 84.7 (80.0-91.0) | <0.001 |
| MAP, mmHg ^c^ | 90.9 (83.3-98.0) | 94.9 (87.7-103.3) | 98.0 (91.8-106.7) | 100.8 (93.3-109.3) | <0.001 |
| TC, mmol/L ^c^ | 4.5 (4.0-5.0) | 4.8 (4.3-5.4) | 5.1 (4.5-5.7) | 5.3 (4.6-6.0) | <0.001 |
| TG, mmol/L ^c^ | 0.7 (0.6-0.8) | 1.0 (0.9-1.2) | 1.5 (1.4-1.8) | 2.9 (2.3-4.2) | <0.001 |
| HDL, mmol/L ^c^ | 1.6 (1.3-1.9) | 1.5 (1.2-1.8) | 1.4 (1.2-1.7) | 1.3 (1.1-1.6) | <0.001 |
| LDL, mmol/L ^c^ | 2.3 (1.8-2.7) | 2.6 (2.2-3.1) | 2.8 (2.3-3.3) | 2.8 (2.1-3.3) | <0.001 |
| TyG index ^a^ | 7.8±0.3 | 8.4±0.1 | 8.8±0.1 | 9.6±0.5 | <0.001 |
| hs-CRP ^a^ | 1.6±3.2 | 1.8±3.2 | 2.1±5.4 | 2.4±3.2 | <0.001 |
| baPWV, cm/s ^a^ | 1335.8±282.6 | 1433.1±292.9 | 1512.8±328.7 | 1566.8±341.1 | <0.001 |
| Diabetes, n (%) ^b^ | 7 (0.2) | 37 (1.2) | 165 (5.2) | 635 (20.0) | <0.001 |
| Hypertension, n (%) ^b^ | 601 (18.9) | 1002 (31.6) | 1369 (43.1) | 1652 (52.0) | <0.001 |
| Note: Q, quartiles; BMI, body mass index; FBG, fasting blood glucose; SBP, systolic blood pressure; DBP, diastolic blood pressure; MAP, mean arterial blood pressure; TC, total cholesterol; TG, fast triglyceride; HDL, high-density lipoprotein; LDL, low-density lipoprotein; TyG index, triglyceride–glucose index; hs-CRP, high-sensitivity C-reactive protein; baPWV, brachial-ankle pulse wave velocity; ^a^, mean ± standard deviation, and the variables were tested by one-way analysis of variance for linear trend; ^b^, the variables were tested by Chi-square test for linear trend; ^c^, median (interquartile range), and the variables were tested by Kruskal–Wallis test. | | | | | |

| **Table S3** Association of TyG index with baPWV in participants with at least two baPWV tests (N=12706) | | | |  |
| --- | --- | --- | --- | --- |
| TyG index | Model 1 | | Model 2 | |
|  | *β*（95% CI） | *P* value | *β*（95% CI） | *P* value |
| baPWV at baseline |  |  |  |  |
| Per 1 unit increase | 74 (68-81) | <0.001 | 37 (31-44) | <0.001 |
| Q1 (5.88-8.14) | Reference | | Reference | |
| Q2 (8.14-8.56) | 38 (25-51) | <0.001 | 17 (5-29) | 0.006 |
| Q3 (8.56-9.06) | 87 (74-100) | <0.001 | 47 (34-59) | <0.001 |
| Q4 (9.06-13.17) | 140 (130-150) | <0.001 | 70 (56-83) | <0.001 |
| baPWV progression |  |  |  |  |
| Per 1 unit increase | 0.53 (0.41-0.65) | <0.001 | 0.33 (0.20-0.46) | <0.001 |
| Q1 (5.88-8.14) | Reference | | Reference | |
| Q2 (8.14-8.56) | 0.25 (0.02-0.49) | 0.037 | 0.13 (-0.11-0.37) | 0.277 |
| Q3 (8.56-9.06) | 0.64 (0.40-0.88) | <0.001 | 0.42 (0.17-0.67) | <0.001 |
| Q4 (9.06-13.17) | 0.88 (0.64-1.10) | <0.001 | 0.48 (0.22-0.74) | <0.001 |
| Note: baPWV, brachial-ankle pulse wave velocity; TyG index, triglyceride–glucose index; Q, quartiles; CI, confidence interval | | | | |
| Model 1, adjusted for age and sex at baseline | |  |  |  |
| Model 2, adjusted for variables in model 1 plus smoking, alcohol drinking, physical activity, MAP, diabetes, hs-CRP, and BMI at baseline | | | | |

| **Table S4** Association of TyG index with arterial stiffness in participants with at least two baPWV tests (N=11071) | | | | | | |
| --- | --- | --- | --- | --- | --- | --- |
| TyG index | Arterial  Stiffness(n) | Incident Rate (per 1000 person-years) | Model 1 | | Model 2 | |
|  |  |  | HR (95%CI) | *P* value | HR (95%CI) | *P* value |
| Overall | 1499 | 33.61 | 1.48 (1.38-1.57) | <0.001 | 1.25 (1.16-1.35) | <0.001 |
| Q1 (5.88-8.11) | 203 | 16.45 | Reference |  | Reference |  |
| Q2 (8.11-8.52) | 318 | 27.46 | 1.28 (1.07-1.53) | 0.006 | 1.13 (0.94-1.35) | 0.190 |
| Q3 (8.52-9.01) | 458 | 43.57 | 1.86 (1.57-2.19) | <0.001 | 1.46 (1.23-1.74) | <0.001 |
| Q4 (9.01-13.17) | 520 | 51.17 | 2.19 (1.86-2.58) | <0.001 | 1.49 (1.25-1.77) | <0.001 |
| Note: TyG index, triglyceride–glucose index; HR, hazard ratio; CI, confidence interval; Q, quartiles | | | | | | |
| Model 1, adjusted for age and sex at baseline | | |  |  |  |  |
| Model 2, adjusted for variables in model 1 plus smoking, alcohol drinking, physical activity, MAP, diabetes, hs-CRP, and BMI at baseline | | | | | | |
